# Inactivation of *Listeria monocytogenes* in ready-to-eat smoked turkey meat by combination with packaging atmosphere, oregano essential oil and cold temperature

**DOI:** 10.1186/s13568-019-0775-8

**Published:** 2019-04-19

**Authors:** Samir A. Mahgoub, Rasha M. El-Mekkawy, Mohamed E. Abd El-Hack, Waleed R. El-Ghareeb, Gamaleldin M. Suliman, Abdullah N. Alowaimer, Ayman A. Swelum

**Affiliations:** 1 Microbiology Department, Faculty of Agriculture, Zagazig University, Zagazig, 44511 Egypt; 2 Botany and Microbiology Department, Faculty of Science, Zagazig University, Zagazig, 44511 Egypt; 3 Poultry Department, Faculty of Agriculture, Zagazig University, Zagazig, 44511 Egypt; 4 Department of Veterinary Public Health and Animal Husbandry, College of Veterinary Medicine, King Faisal University, Hofuf, Saudi Arabia; 5 Food Control Department, Faculty of Veterinary Medicine, Zagazig University, Zagazig, Egypt; 6 Department of Animal Production, College of Food and Agricultural Sciences, King Saud University, P.O Box 2460, Riyadh, 11451 Saudi Arabia; 7 Department of Theriogenology, Faculty of Veterinary Medicine, Zagazig University, Zagazig, 44519 Egypt

**Keywords:** *Listeria monocytogenes*, Smoked-turkey, Oregano, Packaging

## Abstract

The effects of packaging atmosphere, storage temperature and oregano essential oil (EO) on growth of *Listeria monocytogenes* on ready-to-eat smoked turkey were studied. Smoked turkey slices were inoculated with a strain of *Listeria monocytogenes* Scott A (5.95, 5.28 and 5.26 log CFU/g) then vacuum packaged (VP), modified atmosphere packaging (MAP: 40% CO_2_ and 60% N_2_) and MAP with oregano essential oil (MAPEO), respectively. The treated slices were then stored at 0, 5, 10 and 15 °C for 179.88 days and the *L. monocytogenes* Scott A’s growth and microbial shelf life were monitored. The combination of MAP or MAPEO and storage temperature did not allow growth of *L. monocytogenes* higher than log 1 CFU/g during all storage periods. While in VP temperature combinations, the multiplication of bacteria were ≥ 1 log CFU/g. In VP, MAP and MAPEO smoked turkey, the growth of *L. monocytogenes* increased regardless of storage temperature. In MAPEO samples the inoculum in the product was suppressed by ca. 5 log CFU/g at 0, 10 and 15 °C at 180, 117 and 81 days of storage, respectively. The inhibition of *L. monocytogenes* in ready-to-eat smoked turkey by the combinations of MAP and MAPEO was enhanced by storage at 0 or 5 °C. The MAPEO system can be used effectively to control growth of pathogen in processed food when maintaining fixed temperature measures is difficult.

## Introduction

Recently, supplying high quality food is of high consideration in the world (Possas et al. 2017). Concerns are not only about factors such as pesticide and fertilizer use, or contamination with various types of other chemicals (Possas et al. 2017). A major concern is ensuring of food quality and safety with regard to the presence of harmful microorganisms. Many factors have contributed in the incidence the requirement for ethnic imported food, changing of manufacturing process, the expansion of food industry, as well as changing in consumers customs, consumption of refrigerated or frozen ready-to-eat (RTE) food products. Moreover, improvements in retail and distribution practices as centralization of activities, internet shopping and global procurement lead to distant distribution points; hence extended storage periods for a variety of products with variable temperature and humidity requirements. All those lead to great demands for innovation in the field of food packaging industry. Smoked turkey products are ready-to-eat meat products and are commonly sold in vacuum packages with a refrigerated shelf life of 30–60 days. Sliced smoked turkey contains less than 2% salt and fat, pH higher than 6.00 and water activity (a_w_) approximately 0.96. *L. monocytogenes* is a food born pathogen having the ability to survive and grow over harsh conditions such as temperature from zero to 45 °C, pH from 4.5 to 9, and high salt concentration (10% NaCl) (Grau and Vanderlinde 1990a, b). Listeriosis is considered a severe fatal disease in human (Mead 2004).

The critical factors which affect the behaviour of *L. monocytogenes* on modified atmosphere packaged meat include storing temperature, pH, and tissue kind (lean or fat) as well as the background biota had no effect on its growth (Grau and Vanderlinde 1990a, b). After 30 days, no growth of *L. monocytogenes* was detected in turkey roll slices that were previously inoculated with 3 log cfu/g of the pathogen then packaged in high-barrier bags with 70% CO_2_ + 30% N_2_ at either 4 or 10 °C (Farber and Daley 1994). On the other hand, 50:50 mixture of CO_2_:N_2_ showed a lesser inhibition.

In food systems, essential oils (Eos) play an important role as natural antimicrobials which can extend the shelf life of the products (Kalemba and Kunicka 2003; Burt and Reinders 2003; Burt 2004). They exert their effect through decreasing the stabilization of phospholipids bilayer of the cell membrane and modifying membrane permeability. Moreover, plant EOs modify enzymatic system and the genetic material of the bacteria with reduction of ATP production (Lambert et al. 2001; Ultee et al. 2002; Oussalah et al. 2006). The effect of various EOs against *L. monocytogenes* as well as the effect of other environmental factors on such activity have been investigated by Aureli et al. (1992).

Several trials have been done to inhibit the microbial growth in foods, including modifying atmosphere packaging and adding chemical preservatives (Holley and Patel 2005). Usage of an active antimicrobial packaging material is considered promising technique. The evaluation of the effects of vacuum packaged and modified atmosphere with or without essential oil packaged on *L. monocytogenes* inoculated in smoked turkey meat slices stored at 0, 5, 10 and 15 °C was the aim of the present study. Realizing the behaviour of *L. monocytogenes* in different packaging type can afford crucial information that may be helpful to promote the shelf life and safety of minimally processed foods (Holley and Patel 2005).

## Materials and methods

### Sampling procedure

#### Preparation of inoculum and inoculation of smoked turkey

*Listeria monocytogenes* Scott A strain used in this investigation was obtained from Lab. Microbiology and Biotechnology of Foods, Department of Food Science and Human Nutrition, Era Odos 75, Athens 11855, Greece. *L. monocytogenes* Scott A was preserved in broth at − 80 °C. Reactivation of the cultures, before starting our experiments, was made by three successive transfers in tryptic soy broth (Difco Laboratories) at 30 °C for 24 h. Cells were collected by centrifugation (14,000 rpm for 10 min at 4 °C), followed by washing three times and re-suspended in Ringer’s balanced solution (Lab M). The cell numbers were determined by surface plating on polymyxin-acriflavine-lithium chloride-ceftazidime-aesculin-mannitol agar (PALCAM, Biolife) in duplicate. After 48 h of incubation at 30 °C, colonies were counted. A final inoculum was prepared by serial dilution in Ringer’s solution based on the desired cell numbers.

The smoked turkey slices were inoculated with 0.1 ml of 6–7 log CFU/ml populations of *L. monocytogenes.* The final cell count on turkey was ca. 5–6 log CFU/g. The VP smoked turkey samples were opened and after inoculation with the pathogen, or in the case of the control treatment without inoculation with the pathogen, were repackaged with VP, MAP (40% CO_2_ and 60% N_2_) or MAPEO (40% CO_2_ and 60% N_2_ in combination with oregano essential oil of 2%) as described in 2.1.5. Packages from each treatment (VP, MAP or MAPEO) were divided into four groups then each group was stored at 0, 5, 10 or 15 °C and enumerated for *L. monocytogenes* within 6 months.

### Microbiological analysis

For microbiological analyses, 25 g of each sample were transferred aseptically to a stomacher bag (Sewared, London, UK); homogenised with 225 ml of sterile Ringer’s solution (Lab 100 Z) for 60 s with a stomacher (Lab. Blender 400; Seward Medical, London, UK) at room temperature. Decimal solution in Ringer’s solution was prepared. Duplicate 1 ml or 0.1 ml samples of appropriate dilutions were poured or spread on non-selective or selective agar plates.

Determination was carried out as follows: total viable counts (TVC) on plate count agar (PCA; Merck, 1.05463) incubated at 25 °C for 72 h; Lactic Acid Bacilli (LAB) on de Man, Rogosa, Sharpe (MRS Biolife) overlain with 5 ml of the same medium and incubated at 25 °C for 72 h; *Pseudomonas* spp. on cetrimide-fucidin-cephaloridine (CFC) Agar (Lab M, supplemented with selective supplement X109) incubated at 25 °C for 48 h; *B. thermosphacta* on STAA agar (streptomycin sulphate, cycloheximide and thallous acetate agar; Oxoid M881) incubated at 30 °C for 72 h; *Enterobacteriaceae* on Violet Red Bile Glucose Agar (VRBG, Biolife), with a double layer of the same medium incubated at 37 °C for 24 h; enterococci on Kanamycin Aesculin Azide Agar (LAB M 106) incubated at 37 °C for 48 h, counting the brown colonies as enterococci and the black colonies as streptococci and *L. monocytogenes* or *Listeria* spp. on polymyxin-acriflavine-lithium chloride-ceftazidime-aesculin-mannitol agar (PALCAM, Biolife) after incubation at 30 °C for 48 h.

The lowest detection limit of the previous experiments was 2 log_10_ CFU/g except for LAB and *Enterobacteriaceae* for which the limit was 1 log_10_ CFU/g. The bacterial population (by log_10_ CFU/g) is the mean of duplicate plates.

*Technique of Listeria enrichment* The procedure used for isolation of *Listeria* spp. was a two-stage enrichment procedure (ISO 11290). 25 g of sample were transferred aseptically to a stomacher bag; 225 ml of primary enrichment broth (sterile half Fraser broth) were mixed and homogenized for 60 s at room temperature and then incubated at 30 °C for 48 h. Samples (0.1 ml) of primary enrichment cultures was transferred into a secondary enrichment (10 ml sterile Fraser broth). After 24 h of incubation at 30 °C, 0.1 ml or loopful from the secondary enrichment were plated and streak on PALCAM agar at 30 °C for 48 h. Typical colonies of *Listeria* spp. were grown on PLACAM agar for 48 h at 30 °C were 1.5–2 mm in diameter, grey-green colonies with black sunken centres and black halos.

### Identification of Listeria

Tryptone soya agar was used to subculture suspected colonies (Lab. M) and incubated for 24 h at 37 °C. Listeria identification was based on Gram stain, catalase and oxidase reactions, motility at 25 °C, β-haemolysis reaction, and biochemical identification using fermentation of d-xylose, l-rhamnose, α-methyl-d-mannoside and d-mannitol (Allerberger 2003). To identify various fastidious strains, API-*Listeria* was used.

### Measurement of pH and redox potential

The pH and redox potential values of the samples were determined at each sampling interval. Briefly, 25 g of the product sample were blended with 225 ml sterile Ringer’s solution for 60 s. A pH meter (model RL150) and redox model 827 pH Lab (Metrohm) were used for the pH measurement. The redox values were converted by the equation of Midgley and Torrence (1978) were reported as redox potential (mV).

### Measurement of CO_2_ and O_2_

CO_2_ and O_2_ percentages in the MAP and MAPEO were determined using a PBI Dansensor (ChekMate 9900 O_2_/CO_2_).

### Statistical analysis

Obtained data were analysed and processed by Excel and transformed into log CFU/g for all experiments. Analysis of variance (ANOVA) was performed to determine the significant difference (*P *< 0.05) in microbial counts and pH value changes during storage at different temperatures for both types of products. Logarithmic means were separated by Tukey’s multiple range test using SPSS 10.0 for Windows™.

## Results

**Fig. 1:**
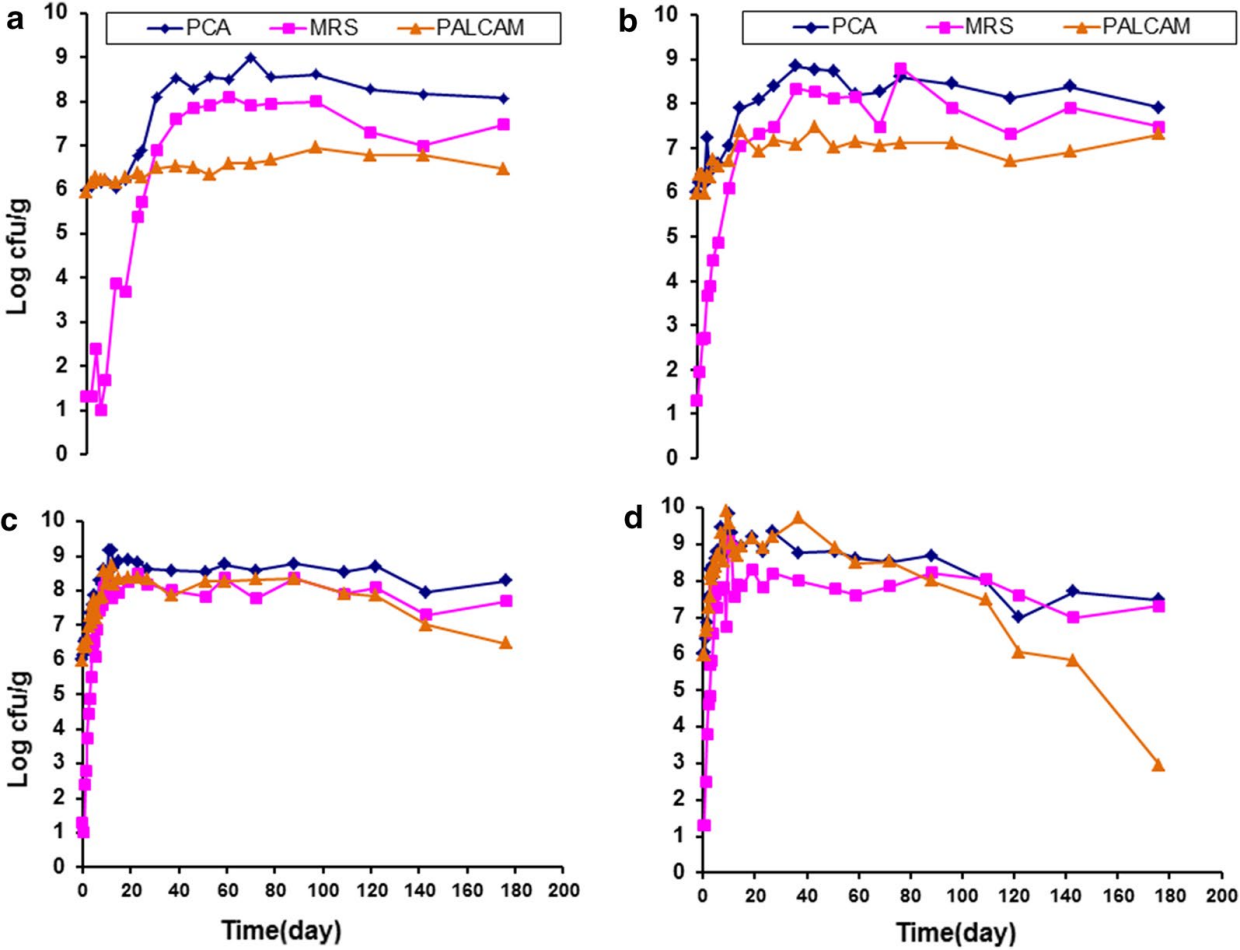
Effect of vacuum packaging and storage temperature (**a** 0 °C; **b** 5 °C; **c** 10 °C and **d** 15 °C) on growth of *Listeria monocytogenes* Scott A (PALCAM) and indigenous microflora (PCA and MRS) in smoked turkey

The effects of vacuum packaging and storage temperature on growth of *Listeria monocytogenes* Scott A (PALCAM) and indigenous microflora (PCA and MRS) in smoked turkey are presented in Fig. 1. Under VP samples (control), the TVC increased to higher than 7 log CFU/g in the smoked turkey slices at 29, 23, 8.0 and 5 days of storage at 0, 5, 10 and 15 °C, respectively.

**Fig. 2:**
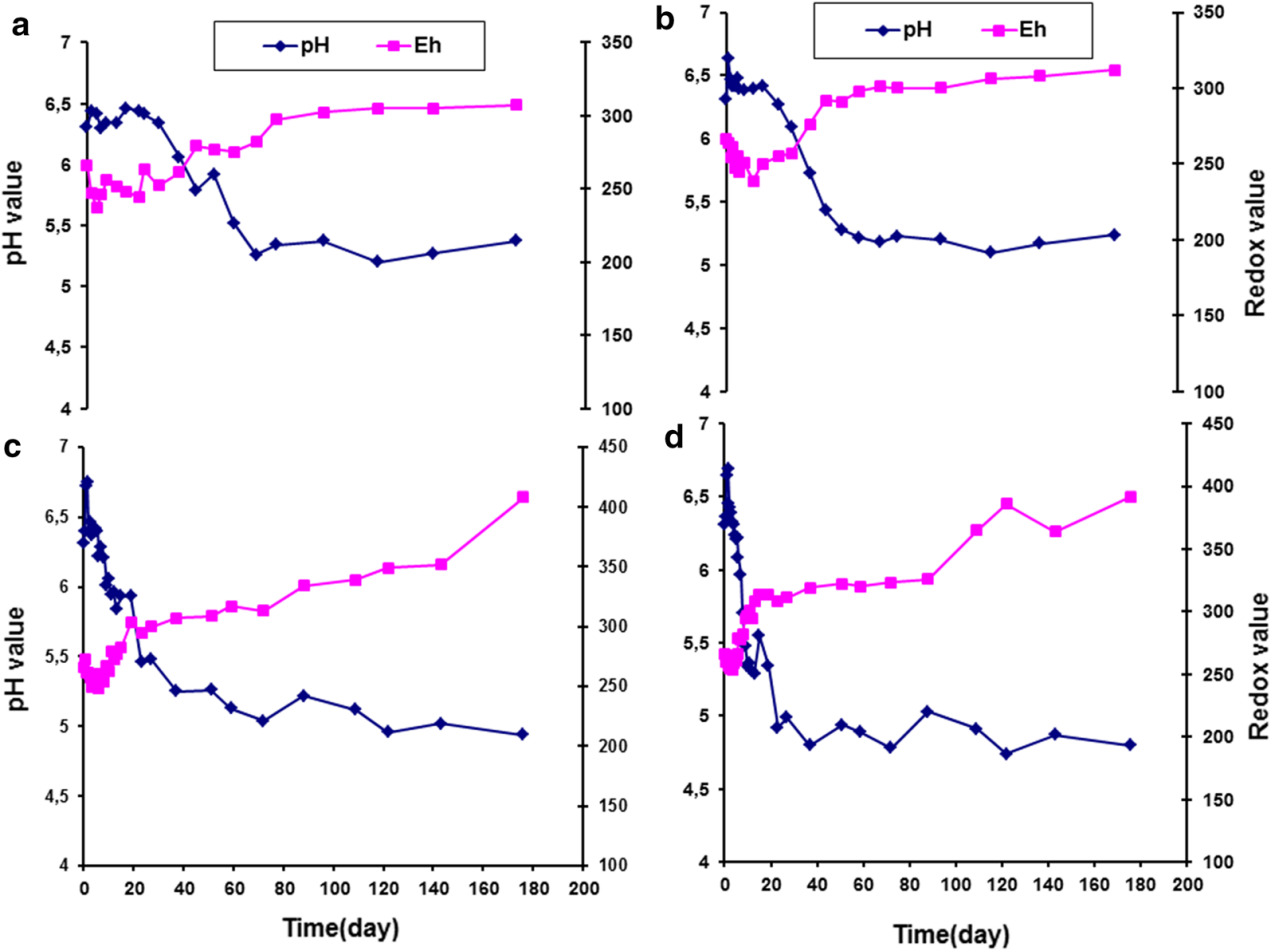
Change of pH and redox values (Eh) of vacuum packaged smoked turkey storage at different temperature (**a** 0 °C; **b** 5 °C; **c** 10 °C and **d** 15 °C) and inoculated with *Listeria monocytogenes* Scott A

**Fig. 3:**
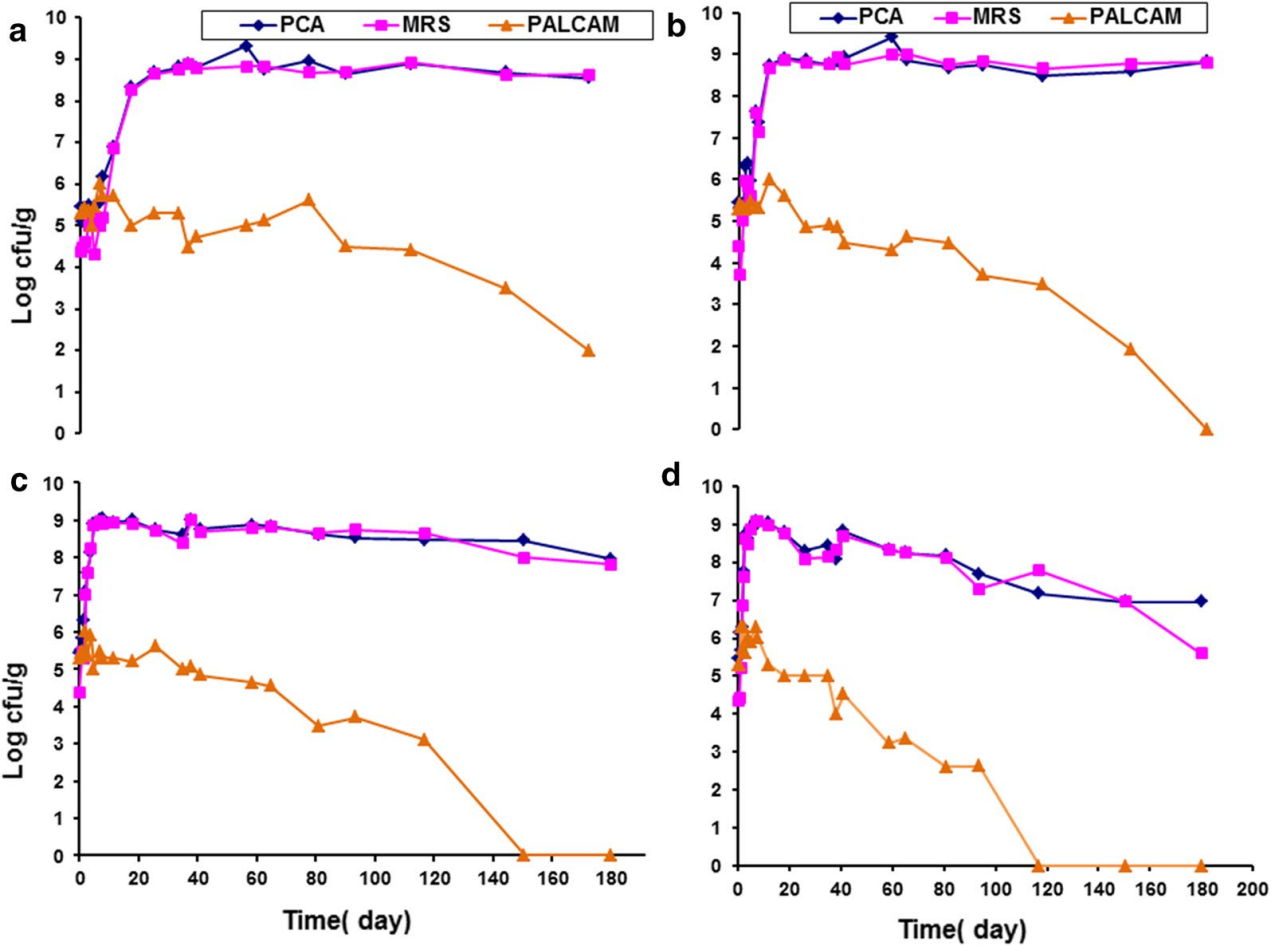
Effect of MAP (40% CO_2_ + 60% N_2_) on growth of indigenous microflora (TVC, total viable count and LAB, lactic acid bacteria in smoked turkey storage at different temperature (**a** 0 °C; **b** 5 °C; **c** 10 °C and **d** 15 °C)

**Fig. 4:**
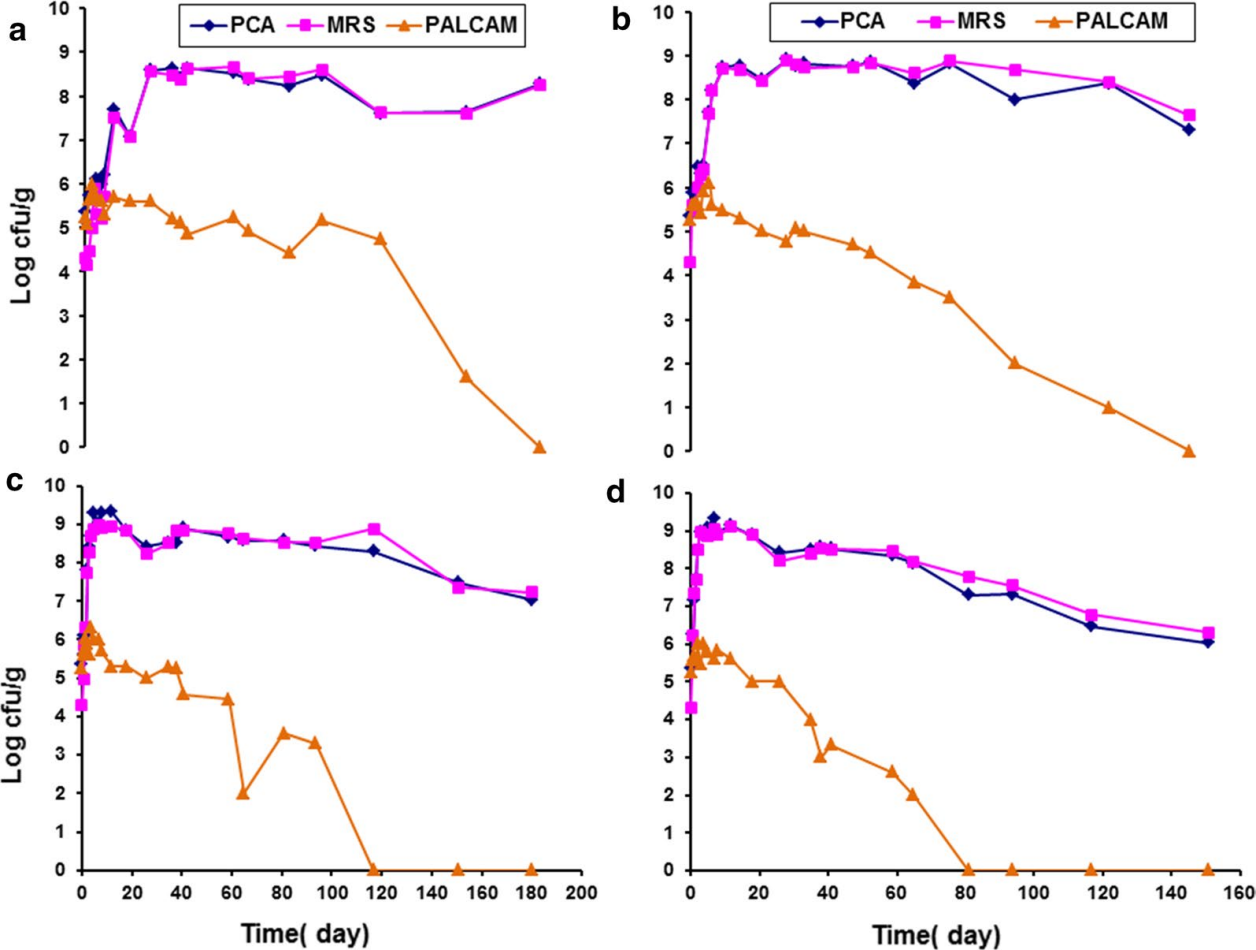
Effect of MAP (40% CO_2_ + 60% N_2_) and essential oil on growth of indigenous microflora (TVC, total viable count and LAB, lactic acid bacteria in smoked turkey storage at different temperature (**a** 0 °C; **b** 5 °C; **c** 10 °C and **d** 15 °C)

**Fig. 5:**
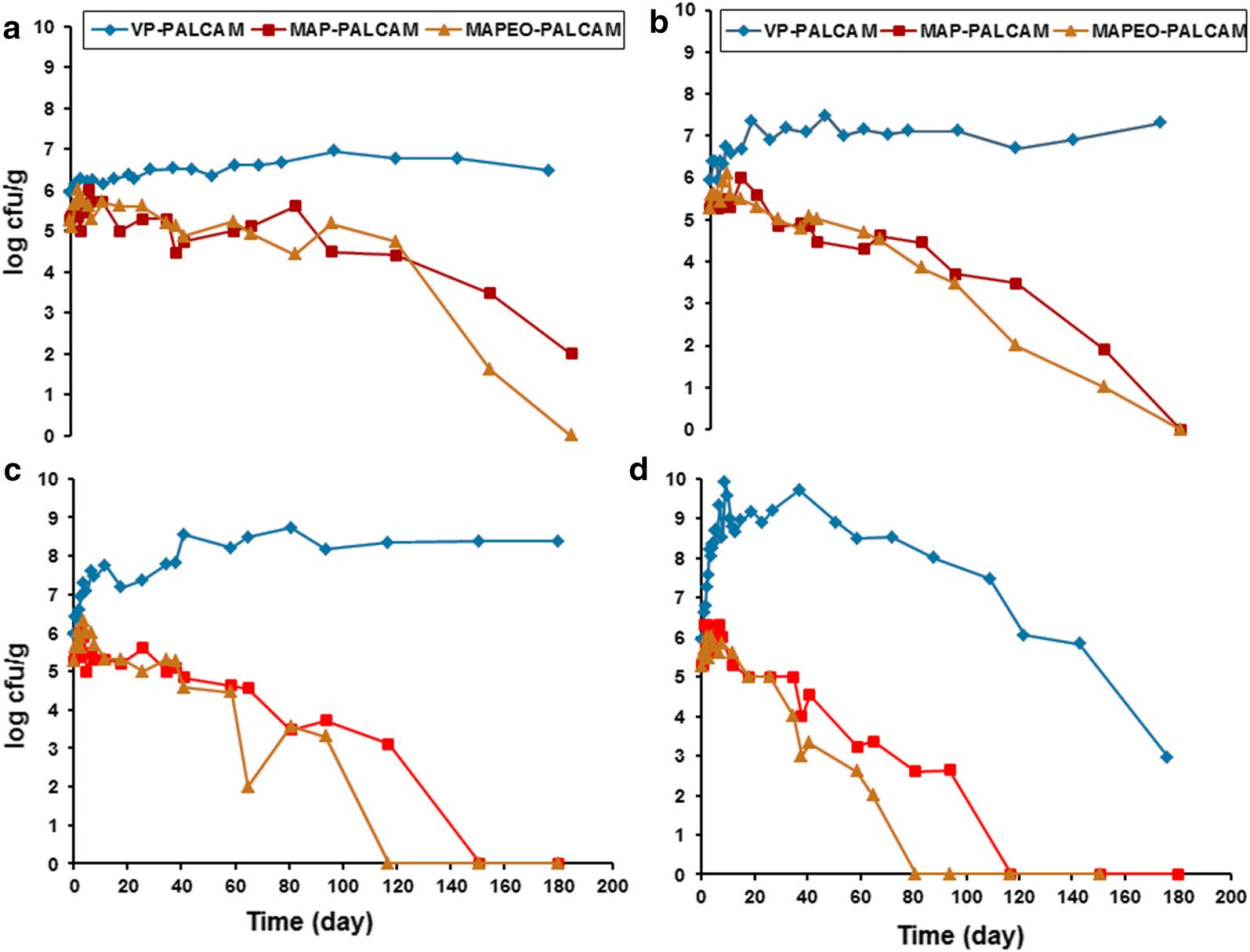
Survival of *Listeria monocytogenes* in VP, MAP and MAP-EO smoked turkey at **a** 0 °C; **b** 5 °C; **c** 10 °C and **d** 15 °C

Figure 2 shows the change in pH and redox values (Eh) of vacuum packaged smoked turkey stored at different temperatures and inoculated with *Listeria monocytogenes* Scott A. The effect of MAP on growth of indigenous microflora in smoked turkey stored at different temperatures is displayed in Fig. 3. Figure 4 shows the effect of MAP and essential oil on growth of indigenous microflora in smoked turkey stored at different temperatures. The survival of *Listeria monocytogenes* in VP, MAP and MAP-EO smoked turkey at (A, 0 °C; B, 5 °C; C, 10 °C and D, 15 °C) is shown in Fig. 5.

**Fig. 6:**
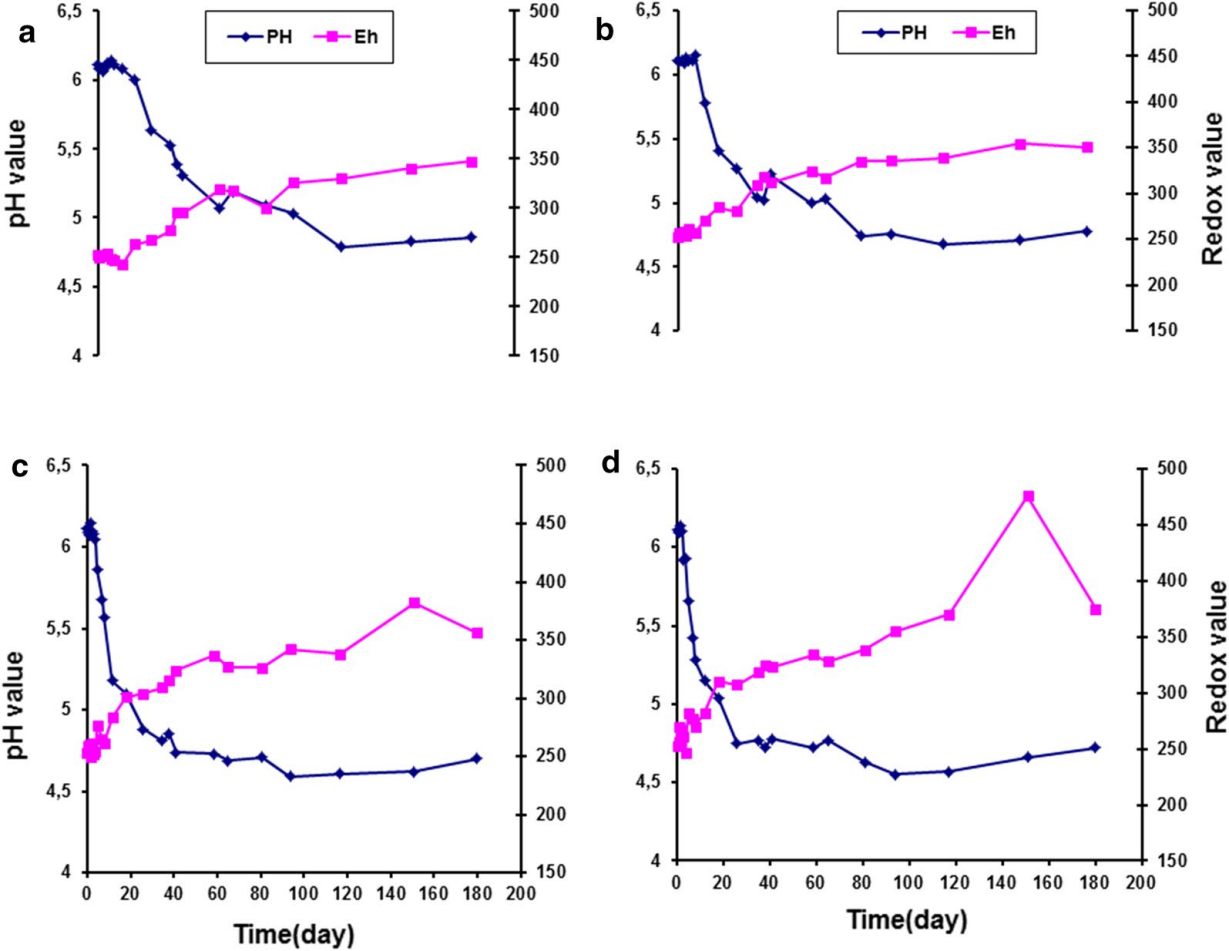
Change of pH and redox values of modified atmosphere packaging smoked turkey without essential oil and stored at **a**, 0 °C; **b** 5 °C; **c** 10 °C and **d** 15 °C

Figure 6 presents the change of pH and redox values of modified atmosphere packaging smoked turkey without essential oil and stored at A, 0 °C; B, 5 °C; C, 10 °C and D, 15 °C.

**Fig. 7:**
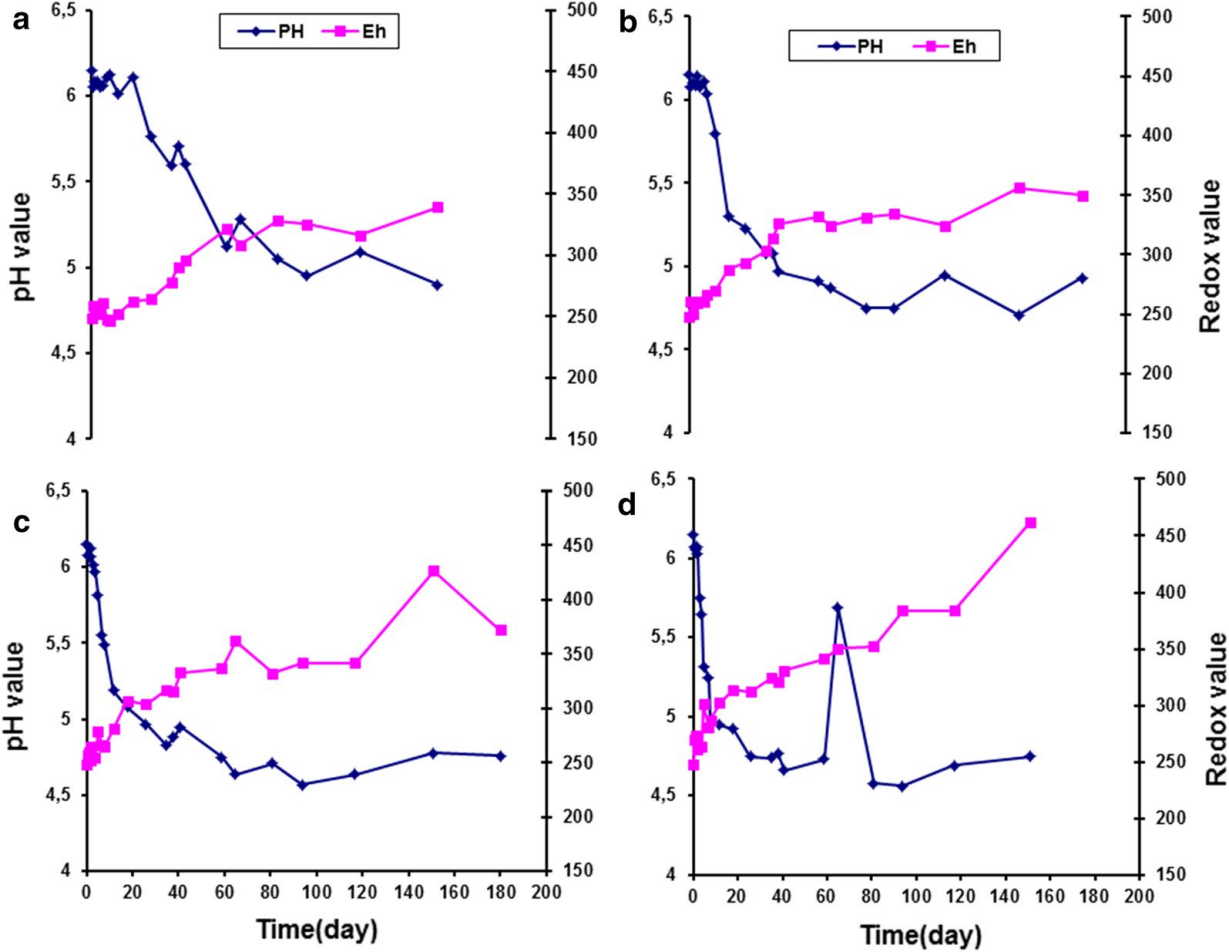
Change of pH and redox values of modified atmosphere packaging smoked turkey with essential oil and stored at **a**, 0 °C; **b**, 5 °C; **c**, 10 °C and **d**, 15 °C

The change of pH and redox values of modified atmosphere packaging smoked turkey with essential oil and stored at A, 0 °C; B, 5 °C; C, 10 °C and D, 15 °C is exhibited in Fig. 7.

## Discussion

### Growth of indigenous flora in vacuum packed smoked turkey stored under different temperatures

The LAB in the control samples steadily increased from 1.3 log CFU/g to the spoilage limit (> 7 log CFU/g) at 50.95, 22.91, 10.5 and 6.5 days of storage at 0, 5, 10 and 15 °C, respectively. In the samples treated with *L. monocytogenes*, the initial TVC in the VP smoked turkey was similar to the pathogen population of ca. 6 log CFU/g. TVC in smoked turkey reached the spoilage limit (> 7 log CFU/g) after 23, 8, 2.5 and 1.5 days of storage at 0, 5, 10 and 15° C, respectively, while the LAB in the samples reached these levels after 29, 12, 6 and 4 days of storage under the same conditions (Fig. 1).

Under MAP and MAPEO, the initial levels of LAB were ca. 5 log CFU/g. The LAB had similar patterns growth under MAP and MAPEO, and reached the spoilage limit after 11.75, 4.75, 1.63 and 0.63 days of storage at 0, 5 10 and 15 °C, respectively (Figs. 3, 4).

### Changes in the pH and redox values in smoked turkey under different storage temperatures

The evaluation of pH and redox potential in smoked turkey showed similar patterns under the three packaging combinations and different temperature regimes. A slight change in pH and redox potential was observed during the shelf life of the product in all of the control and treated smoked turkey samples. At the end of storage, a high drop in the pH and high increase in redox potential were observed under all storage temperatures and packaging conditions (Figs. 3, 4, 6, 7).

### Changes in gases in MAP and MAPEO samples

The concentration of gases in the packages throughout the storage period showed only little change from the beginning until the end of storage, highlighting the effectiveness of the mechanical barriers for gas exchange.

### The behaviour of *L. monocytogenes* in ready-to-eat (RTE) smoked turkey

From the control experiment on smoked turkey (without inoculation of *L. monocytogenes* Scott A), no *L. monocytogenes* were found throughout the period of storage (96 days) at 0, 5, 10 and 15 °C, which demonstrate that *L. monocytogenes* was likely not initially present in the smoked turkey used. During the shelf life of the product inoculated with *L. monocytogenes*, the maximum increases in the growth of *L. monocytogenes* were by 0.54, 0.72 and 0.74 log CFU/g at 0 °C, 0.78, 0.20 and 0.64 log CFU/g at 5 °C, 1.33; 0.72 and 0.78 log CFU/g at 10 °C; and 2.41, 1.02 and 0.32 log CFU/g) at 15 °C, under packaging with VP, MAP and MAPEO, respectively (Fig. 5). The application of MAP and MAPEO is within the U.S. Department of Agriculture, Food Safety and Inspection Service (2004) guidelines as it did not allow the pathogen to increase by more than 1 log CFU/g during the shelf life of the product, but the application of VP to samples did allow such an increase.

### Under vacuum packaging

The initial level of the pathogen in the VP smoked turkey was 5.95 log CFU/g. The levels of *L. monocytogenes* increased to ≥ 7 log CFU/g after 76, 23, 2.5 and 1.5 days of storage at 0, 5, 10 and 15 °C, respectively. Storage at 0 and 5 °C allowed slower growth of *L. monocytogenes* than at 10 and 15 °C, but it did not allow the pathogen to increase more than 1 log CFU/g, while storage at 10 and 15 °C allowed the pathogen to increase by ca. 2 log CFU/g during the shelf life of smoked turkey. The ability of *L. monocytogenes* to grow on VP raw and industrially processed (frankfurters) beef and pork meat has been documented by other studies (Grau and Vanderlinde 1990a, b; Porto et al. 2002; Samelis et al. 2002). Therefore, a combination of low-temperature storage in the presence of packaging film was found to provide suitable condition for the survival of *L. monocytogenes* on smoked turkey for a period longer than 5 months. Another study indicated that VP enhances the survival of *L. monocytogenes* on meat products compared to aerobic storage (unpackaged products), especially at low temperatures (Gounadaki et al. 2007).

### Under MAP

The initial levels of the pathogen in the smoked turkey were 5.28 log CFU/g. Indeed, MAP smoked turkey did not allow the pathogen to increase in the product by > 1 log CFU/g throughout the period of storage under all storage conditions. Rutherford et al. (2007) reported that CO_2_ packaging of ready-to-eat shrimp and storage at 3 °C not only had a great influence on growth of *L. monocytogenes* and other psychrotrophic bacteria but also increased the shelf life in comparison to vacuum packaging. In addition, storage temperatures higher than 3.3 °C did not inhibit the pathogen. The combination of MAP with all the storage regimes seemed to effectively inhibit growth and cause the death of *L. monocytogenes*, whose numbers decreased by > 1 log CFU/g after 116.83, 80.83, 64.83 and 40.83 days of storage at 0, 5, 10 and 15 °C, respectively. Carbon dioxide inhibition of *L. monocytogenes* is effective even at abusive temperatures as illustrated by Avery et al. (1995).

### Under MAP with oregano essential oil

The initial level of *L. monocytogenes* Scott A in the smoked turkey was 5.26 log CFU/g. The MAPEO samples under different storage temperatures did not permit the growth of *L. monocytogenes* by more than log 1 CFU/g throughout the period of storage. Under MAPEO, the inoculum in the product was suppressed by ca. 5 log CFU/g (0 and 5), 10 and 15 °C after (150.79 days), 93.71 and 64.83 days of storage, respectively. A strong decrease in viable cells was noticed under MAPEO compared to MAP and VP under all temperatures. In another study by Mytle et al. (2006) on RTE frankfurters and clove essential oil, Scott A exhibited growth of 1.0 log_10_ CFU/g in 14 days when a high inoculum of 6.0 log_10_ CFU/g was applied at 5 °C. In contrast, Hao et al. (1998a) observed no growth at high populations of *L. monocytogenes* (Scott A) in the controls at 5 °C. Mytle et al. (2006) stated that the essential oil treatments exhibited a reduction in final *L. monocytogenes* populations at 5 °C and 15 °C storage as compared to the control in all treatments including Scott A. The essential oils do have greater effect in broth culture medium than in food as observed by Hao et al. (1998b).

Results assured that the inhibition of *L. monocytogenes* in RTE smoked turkey under MAP and MAPEO (at 0 and 5 °C) was enhanced compared to VP during the shelf life of the product. The MAPEO system could be efficiently used to control the growth of the pathogen when strict temperature control is difficult as during processing, transportation, retail display, or home use. By contrast, VP under different temperatures allowed for multiplication of the pathogen, with 1–3 log_10_ CFU/g.
